# Ruptured Splenic Artery Aneurysm in the Postpartum Patient: A Case Series

**DOI:** 10.5811/cpcem.2020.4.46946

**Published:** 2020-07-15

**Authors:** Angel Rochester, Tracy Lance, Dane E. Smith, Camiron Pfennig, Adam Tyson, Phillip Moschella

**Affiliations:** * Prisma Health Upstate, Department of Emergency Medicine, Greenville, South Carolina; † Prisma Health Upstate, Department of Surgery, Greenville, South Carolina; ‡ Prisma Health Upstate, Department of Obstetrics and Gynecology, Greenville, South Carolina

**Keywords:** splenic artery aneurysm, postpartum complications, antepartum complications

## Abstract

**Introduction:**

The evaluation of an unstable peripartum patient in the emergency department includes a differential diagnosis spanning multiple organ systems. Splenic artery aneurysm (SAA) is one of those rare diagnoses with potentially high morbidity and mortality.

**Case Series:**

This case series explores two unusual cases of postpartum SAAs. Despite differences in presentation, both patients had a ruptured SAA.

**Conclusion:**

Often, SAAs are misdiagnosed. Early diagnosis is key, especially for the fetus. If the patient presents in shock, the expedited diagnosis and treatment can be lifesaving for both the mother and the fetus.

## INTRODUCTION

The evaluation of an unstable pregnant or postpartum patient includes vast differential diagnoses that span multiple organ systems. Splenic artery aneurysm (SAA) is a dilation in a focal area of the splenic artery leading to potential instability. A rare diagnosis with high morbidity and mortality that is not well understood or recognized in the literature, SAA should be included in the discussion of potential pathology in this patient population, especially by emergency physicians. Often, SAAs are missed or misdiagnosed and found only in the operating room after rupture or in autopsy, with necropsy rates of as high as 10%.^6^,^8^ The expedited diagnosis and subsequent repair or embolization can be lifesaving for both the mother and the fetus. This report highlights a small case series of patients to display the varying presentations of SAAs, and the diagnostic and treatment options associated with this disease.

## CASE SERIES

### Case 1

A 29-year-old gravida 1 para 1 female, postpartum day four from an uncomplicated spontaneous vaginal delivery, arrived at the emergency department (ED) by emergency medical services, unstable but awake, complaining of abdominal pain that started 24 hours prior. She was initially found by paramedics on the bathroom floor awake with an initial blood pressure of 60/40 millimeters of mercury (mmHg); thus, an intravenous (IV) catheter was placed and the patient received a 1000 milliliter (mL) bolus of normal saline. On arrival to the ED, the patient was pale, diaphoretic, and actively vomiting with a heart rate of 134 beats per minute, temperature 36.8 degrees Celsius (°C) (98.2 degrees Fahrenheit [°F]), respirations of 28 per minute, and a blood pressure of 84/31 mmHg.

A focused assessment with sonography in trauma (FAST) exam was performed and showed a significant amount of fluid across all abdominal views. The patient’s initial lactic acid was 9.5 millimoles per liter (mmol/L) (reference range 0.5 – 2.5 mmol/L) and her hemoglobin was 5 grams per deciliter (g/dL) (reference range 14 – 18 g/dL). At this point both the general surgery and obstetrics/gynecology services were consulted. Point-of-care ultrasonography (POCUS) performed by obstetrics showed no abnormalities of the uterus. The patient’s declining hemodynamic status necessitated transfusion, and she received four units of packed red blood cells and one unit of fresh frozen plasma. Despite this aggressive resuscitation, the patient’s mental status declined requiring intubation for airway protection, and she was taken by both general surgery and obstetrics for emergency exploratory laparotomy. During the exploratory laparotomy, she was found to have a hemorrhage secondary to a ruptured SAA. The patient was managed laparoscopically with suture ligation of the SAA and a splenectomy. Her postoperative recovery was uneventful. She was discharged on day three postoperatively, and at her six-month follow-up she remained healthy with no complications.

### Case 2

A 46-year-old gravida 11 para 9 woman with history of hypertension and an uncomplicated caesarean section four months prior arrived complaining of sudden onset of mid to lower back pain and cramping that radiated to her upper abdomen and chest with associated shortness of breath with exertion. Physical exam showed a tachycardic, moderately anxious female without back or abdominal tenderness on palpation. Her initial vital signs were documented as follows: heart rate 130 beats per minute; blood pressure 116/82 mmHg; respiratory rate 16 breaths per minute; temperature 36.3°C (97.4°F); and O_2_ saturation 100%.

A FAST exam was negative for free fluid, and her initial labs were unremarkable. Despite fluid resuscitation, she remained tachycardic. Blood pressure readings were obtained in both of her upper extremities and a 20 mmHg difference was noted, increasing the concern for dissecting aortic aneurysm. A computed tomography (CT) of the chest with IV contrast showed a splenic abnormality vs colonic gas. At that time, a subsequent CT of abdomen and pelvis with oral contrast was obtained that revealed a large amount of fluid within the greater peritoneal cavity and lesser sac. General surgery was consulted and vascular SAA was identified and coiled. During her hospital course she remained stable. She was discharged on day three postoperatively and has not had any other complications to date.

## DISCUSSION

SAAs, first reported in 1770, are the most common visceral aneurysm and the third most common intra-abdominal aneurysm, behind those affecting the aorta and iliac artery.^1^ Typically, unruptured SAAs are asymptomatic; but occasionally they will present with vague complaints including abdominal pain. A ruptured SAA is often fatal and therefore must be included in your differential of abdominal pain. The general population has a prevalence of 0.78%, with a female predominance of 4:1 and necropsy rates as high as 10%.^6^,^8^ Risk factors for a SAA include female sex, pregnancy, multiparity, portal hypertension (cirrhosis and liver transplant), collagen vascular disease, medial fibrodysplasia, atherosclerosis, and splenomegaly.^5^,^8^

**CPC-EM Capsule**

What do we already know about this clinical entity?

Splenic artery aneurysm (SAA) is an often overlooked and unrecognized diagnosis with high mortality, especially in women.

What makes this presentation of disease reportable?

Two postpartum patients one week and three months postpartum: Both were unstable, but rapid diagnosis and interventions led to good clinical outcomes.

What is the major learning point?

Pregnancy increases the development of SAAs that could rupture at any time in the life of a woman. When they rupture, there is a “double rupture” phenomenon.

How might this improve emergency medicine practice?

Awareness of this rare clinical entity could spur more rapid diagnosis to prevent significant morbidity and mortality to one or possibly two lives, mother and baby.

Both patients in our cases likely developed SAAs during their pregnancies. In fact, of the more than 400 cases reported in the international literature of ruptured SAAs, 30% occurred during pregnancy, and 6% in the postpartum phase.^2^,^3^ Multiparity is a strong risk factor with a mean of 3.5 pregnancies.^2^,^4^ The risk factors for rupture include aneurysm size greater than two centimeters (cm), female of childbearing age, pregnancy, cirrhosis, liver transplant, and alpha-1 antitrypsin deficiency.^5^,^7^

The association with pregnancy is not understood, but changes related to the hormones and hemodynamics likely contribute. Estrogen, progesterone, and relaxin have vasodilatory effects and can increase the compliance and elasticity of vessel walls resulting in evidence of elastin formation failure, disruption of the internal elastic lamina, and elastic fiber fragmentation as a result of the elevated hormone levels. The hormones also cause degeneration of smooth muscle. Hemodynamically, the enlarging uterus compresses the surrounding vascular structures leading to higher pressure and flow in the splenic artery. This hemodynamic change is secondary to higher blood volumes, as the plasma portion is increased by up to 50%; increased cardiac output; and relative portal congestion.

The generalized risk of splenic rupture is approximately 5%, but this is increased in pregnancy and with larger overall aneurysm size greater than 2 cm.^5^,^7^ The mortality secondary to splenic rupture is 25–36%, but increases to 75% if the patient is pregnant.^6^ If found during pregnancy, the risk rises to 95% rupture with a fetal mortality of 95%.^6^ Splenic rupture can occur at any time in pregnancy, including the postpartum period, which is less common but possible, as highlighted in both of these cases.

SAAs are difficult to diagnose, especially if asymptomatic. If symptomatic, patients usually have vague complaints of abdominal, back, or chest pain with radiation to the left shoulder. In pregnancy, SAAs are misdiagnosed as uterine rupture up to 70% of the time.^6^ Other common diagnoses include ectopic pregnancy, placental abruption, amniotic fluid embolism, and perforated ulcer.

Approximately 25% of SAA ruptures are described in the literature as the “double rupture” phenomenon.^7^–^9^ Both cases are consistent with this phenomenon as the initial rupture spills into the lesser sac, which is posterior to the stomach and lesser omentum, leading only to mild symptoms as the local anatomic structures tamponades the hemorrhage in this area. Once a critical volume is reached, the hemorrhage spills through the foramen of Winslow into the greater sac (the larger portion of the peritoneal cavity) resulting in severe symptoms and hemodynamic instability, which often evolves within 6–96 hours after initial symptom onset.^7^–^9^ During pregnancy, hypotension may not be evident until approximately 35% of the circulating blood volume is lost making high clinical suspicion required for improved outcomes.

Although our patient in case one did not suffer any trauma, a FAST exam was instrumental in rapid diagnosis and treatment as it demonstrated free fluid in the hepatorenal, splenorenal, and pelvic views. The FAST exam in case two was likely “negative” because of the known limits of detection for free fluid for this exam and the “double rupture” phenomenon.

SAAs are treated either endovascularly or surgically. If unruptured or uncomplicated, then endovascular embolization is preferred. If rupture is suspected, an emergent exploratory laparotomy is indicated. Since 80% of these aneurysms are located distally (Image), the treatment is usually resection and splenectomy.^1^,^8^

## CONCLUSION

Although rare, splenic artery aneurysms should be included in the differential diagnosis of abdominal pain, especially in the unstable peripartum patient. The risk of rupture is 5% but increases with pregnancy and can have a mortality rate of up to 75%.^6^ Emergency physicians should consider splenic artery aneurysm early in the patient evaluation and use varied imaging modalities (i.e., POCUS or CT) to aid in rapid evaluation and subsequent consultation for definitive management to improve outcomes for both the mother and the fetus.

## Figures and Tables

**Image:**
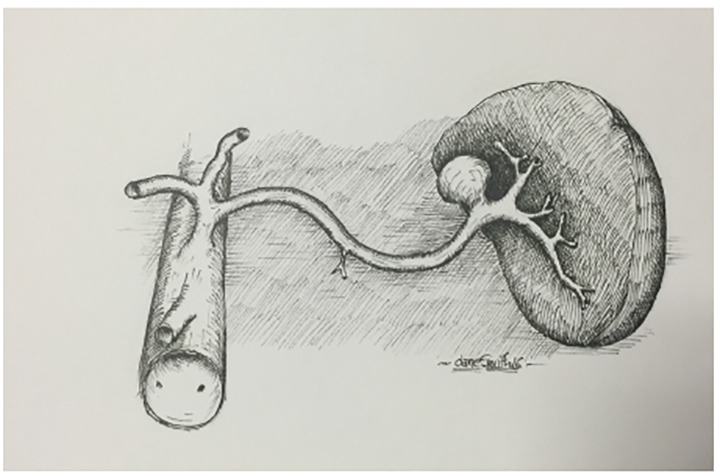
Drawing of a distal splenic artery aneurysm under the spleen. Artist: Dane Smith.
